# Supplementary material for: Generic approach for mathematical model of multi-strain pandemics
Source: PLoS One. 2022 Apr 28;17(4):e0260683. doi: 10.1371/journal.pone.0260683 (PMC9049317; doi:10.1371/journal.pone.0260683)
Supplement: S1 Appendix — (PDF) [file pone.0260683.s001.pdf]

Generic Approach For Mathematical Model of Multi-Strain Pandemics

Teddy Lazebnik[1] and Svetlana Bunimovich-Mendrazitsky[2]

[1]Department of Cancer Biology, Cancer Institute, University College London, UK
[2]Department of Mathematics, Ariel University, Israel

1 Model's development $|M| = 2$

In Eq. (1), $\frac{dR_\emptyset I_1(t)}{dt}$ is the dynamical amount of individuals that did not recovered yet and are infected with the strain ($i = 1$) over time.

$$\frac{dR_\emptyset I_1(t)}{dt} = \beta_{\emptyset,1}(R_\emptyset I_1(t) + R_{\{2\}} I_1(t))R_\emptyset(t) - \gamma_{\emptyset,1} R_\emptyset I_1(t), \quad (1)$$

In Eq. (2), $\frac{dR_{\{2\}} I_1(t)}{dt}$ is the dynamical amount of suspected individuals that recovered from the strain ($i = 2$) and are infected with the strain ($i = 1$) over time.

$$\frac{dR_{\{2\}} I_1(t)}{dt} = \beta_{\{2\},1}(R_{\{2\}} I_1(t) + R_\emptyset I_1(t))R_{\{2\}}(t) - \gamma_{\{2\},1} R_{\{2\}} I_1(t), \quad (2)$$

In Eq. (3), $\frac{dR_\emptyset I_2(t)}{dt}$ is the dynamical amount of suspicious individuals that are infected by the strain ($i = 2$) over time.

$$\frac{dR_\emptyset I_2(t)}{dt} = \beta_{\emptyset,2}(R_\emptyset I_2(t) + R_{\{1\}} I_2(t))R_\emptyset(t) - \gamma_{\emptyset,2} R_\emptyset I_2(t), \quad (3)$$

In Eq. (4), $\frac{dR_{\{1\}} I_2(t)}{dt}$ is the dynamical amount of individuals that recovered from the strain ($i = 1$) and infected with the strain ($i = 2$) over time.

$$\frac{dR_{\{1\}} I_2(t)}{dt} = \beta_{\{1\},2}(R_{\{1\}} I_2(t) + R_\emptyset I_2(t))R_{\{1\}}(t) - \gamma_{\{1\},2} R_{\{1\}} I_2(t), \quad (4)$$

In Eq. (5), $\frac{dR_\emptyset(t)}{dt}$ is the dynamical amount of individuals that did not get infected did they recover or not recover.

$$\frac{dR_\emptyset(t)}{dt} = -R_\emptyset(t)\big(\beta_{\emptyset,1}(R_\emptyset I_1(t) + R_{\{2\}} I_1(t)) + \beta_{\emptyset,2}(R_\emptyset I_2(t) + R_{\{1\}} I_2(t))\big), \quad (5)$$

In Eq. (6), $\frac{dR_{\{1\}}(t)}{dt}$ is the dynamical amount of individual that recovered from the strain ($i = 1$) over time.

$$\frac{dR_{\{1\}}(t)}{dt} = \gamma_{\emptyset,1}\phi_{\emptyset,1} R_\emptyset I_1(t) - \beta_{\{1\},2}(R_{\{1\}} I_2(t) + R_\emptyset I_2(t))R_{\{1\}}(t), \quad (6)$$

In Eq. (7), $\frac{dR_{\{2\}}(t)}{dt}$ is the dynamical amount of individual that recovered from the strain ($i = 2$) over time.

$$\frac{dR_{\{2\}}(t)}{dt} = \gamma_{\emptyset,2}\phi_{\emptyset,2} R_\emptyset I_2(t) - \beta_{\{2\},1}(R_{\{2\}} I_1(t) + R_\emptyset I_1(t))R_{\{2\}}(t), \quad (7)$$

In Eq. (8), $\frac{dR_{\{1,2\}}(t)}{dt}$ is the dynamical amount of individual that recovered from both the strains ($i \in \{1, 2\}$) over time.

$$\frac{dR_{\{1,2\}}(t)}{dt} = \gamma_{\{2\},1}\phi_{\{2\},1}R_{\{2\}}I_1(t) + \gamma_{\{1\},2}\phi_{\{1\},2}R_{\{1\}}I_2(t), \tag{8}$$

In Eq. (9), $\frac{dD(t)}{dt}$ is the dynamical amount of individuals that die due to the pandemic over time.

$$\begin{aligned}\frac{dD(t)}{dt} &= \gamma_{\emptyset,1}(1-\phi_{\emptyset,1})R_{\emptyset}I_1(t) + \gamma_{\{2\},1}(1-\phi_{\{2\},1})R_{\{2\}}I_1(t)\\ &+\gamma_{\emptyset,2}(1-\phi_{\emptyset,2})R_{\emptyset}I_2(t) + \gamma_{\{1\},2}(1-\phi_{\{1\},2})R_{\{1\}}I_2(t).\end{aligned} \tag{9}$$

2 The proposed model reduced to the SIRD model

The proposed multi-strain model is isomorphic to the *SIRD* model [1] in the case of $|M| = 1$. Recall, the *SIRD* model is represented using the following system of ordinary differential equations [1]:

$$\begin{aligned}\tfrac{dS(t)}{dt} &= -\beta I(t)S(t),\\ \tfrac{dI(t)}{dt} &= \beta I(t)S(t) - \gamma I(t),\\ \tfrac{dR(t)}{dt} &= \gamma\rho I(t)\\ \tfrac{dD(t)}{dt} &= \gamma(1-\rho)I(t),\end{aligned}$$

where (t) is the portion of susceptible individuals from the population over time, $I(t)$ is the portion of infected individuals from the population over time, $R(t)$ is the portion of recovered individuals from the population, and $D(t)$ is the portion of individuals that die due to the pandemic from the population.

On the other hand, by setting $|M| = 1$ in Eq. (4), one obtains the following system of equations. From Eq. (1), and due to the fact there is only one strain, the group of recovered strain J is empty ($J = \emptyset$). As such, an individual can be infected by one strain 1 while not recovering from other (as they are non-what). Therefore,

$$\frac{dR_{\emptyset}I_1(t)}{dt} = -\gamma_{\emptyset,1}R_{\emptyset}I_1(t) + \beta_{\emptyset,1}R_{\emptyset}(t)R_{\emptyset}I_1(t).$$

Furthermore, from Eq. (2), one obtains two equations: the group of recovered strain J is empty ($J = \emptyset$) and where the individuals recovered from the one strain ($i = 1$) $J = \{1\}$. Therefore,

$$\begin{aligned}\tfrac{dR_{\emptyset}(t)}{dt} &= -\beta_{\emptyset,1}R_{\emptyset}(t)R_{\emptyset}I_1(t),\\ \tfrac{dR_{\{1\}}(t)}{dt} &= \gamma_{\emptyset,1}\rho_{\emptyset,1}R_{\emptyset}I_1(t).\end{aligned}$$

In addition, from Eq. (3), as the sum $i \in M$ takes the form $i \in \{1\}$, and since the group $j = M\backslash\{i\}$, one obtains:

$$\frac{dD(t)}{dt} = \gamma_{\emptyset,1}(1-\rho_{\emptyset,1})R_{\emptyset}I_1(t).$$

In summary, Eq. (4) for $|M| = 1$ takes the form:

$$\begin{aligned}\tfrac{dR_{\emptyset}I_1(t)}{dt} &= -\gamma_{\emptyset,1}R_{\emptyset}I_1(t) + \beta_{\emptyset,1}R_{\emptyset}(t)R_{\emptyset}I_1(t).\\ \tfrac{dR_{\emptyset}(t)}{dt} &= -\beta_{\emptyset,1}R_{\emptyset}(t)R_{\emptyset}I_1(t),\\ \tfrac{dR_{\{1\}}(t)}{dt} &= \gamma_{\emptyset,1}\rho_{\emptyset,1}R_{\emptyset}I_1(t),\\ \tfrac{dD(t)}{dt} &= \gamma_{\emptyset,1}(1-\rho_{\emptyset,1})R_{\emptyset}I_1(t).\end{aligned}$$

Technically, we perform the following annotation replacement:

$$R_{\emptyset}I_1 = I,\ R_{\emptyset} = S,\ R_{\{1\}} = R,\ \beta_{\emptyset,1} = \beta,\ \gamma_{\emptyset,1} = \gamma,\ \rho_{\emptyset,1} = \rho.$$

The careful reader is able to notice that the two systems of equations are identical. Hence, the proposed model for $|M| = 1$ is isomorphic to the *SIRD* model.

References

[1] M. Al-Raeei. The forecasting of covid-19 with mortality using SIRD epidemic model for the united states, russia, china, and the syrian arab republic. *AUO Advances*, 10(6), 2020.
