# Supplementary material for: Generic approach for mathematical model of multi-strain pandemics
Source: PLoS One. 2022 Apr 28;17(4):e0260683. doi: 10.1371/journal.pone.0260683 (PMC9049317; doi:10.1371/journal.pone.0260683)
Supplement: Attachment — Submitted filename: answer_letter.pdf [file pone.0260683.s002.pdf]

First of all, we would like to thank the editor and the reviews for the careful review and the thought-provoking comments.

Editor

Comment 1: “Please ensure that your manuscript meets PLOS ONE's style requirements, including those for file naming. The PLOS ONE style templates can be found at “

Answer 1: We used PLOS ONE’s style guide, we hope now it is fine now.

Comment 2: “Please include your amended statements within your cover letter; we will change the online submission form on your behalf.”

Answer 2: We add the “The authors received no specific funding for this work.” statement to the cover letter.

Comment 3: “We note that you have stated that you will provide repository information for your data at acceptance.”

Answer 3: All the relevant data used in this research is publicly available - we cited the sources in the manuscript.

Comment 4: “One of the noted authors is a group or consortium [Lorem Ipsum Consortium]. “

Answer 4: We apologize, this is just a technical mistake due to the placeholder in the template. Of course, there is no author “Lorem Ipsum Consortium”.

Comment 5: “ Please include captions for your Supporting Information files at the end of your manuscript, and update any in-text citations to match accordingly. “

Answer 5: We include the supporting information files at the end of the manuscript.

Reviewer #1

Comment 1: “The authors present a multi-strain epidemic model that, albeit quite complicated, does not address basic issues in an epidemic: such as

reinfection and latency periods, which for example play a foremost role in the current COVID-19 epidemic.”

Answer 1: The reviewer points out important epidemiological processes that are missing from the model. Indeed, the proposed model does not address the latency periods (commonly known as the exposed state) since the model, as stated by the reviewer, is complex already, and adding Expose State introduces an additional level of complexity that we were trying to avoid. Regarding the reinfection, the model allows an individual in the population to be reinfected by other strains of the pandemic, as shown in figure 1. Other models allow reinfection of the same strain such as the SIS model (Reluga, 2008) but our model is based on the SIR model, which is a valid epidemiological model, used in an endless number of works (Oka et al., 2020; Cooper et al., 2020; Khyar and Allali, 2020), obtaining wonderful results on a wide range of epidemiological scenarios. Moreover, reinfection from the same strain is valid for the COVID-19 but not for all types of pandemics. For example, HIV is considered a pandemic but does not have reinfection (Quinn, T. C. Global burden of the HIV pandemic. Lancet, 1996.) of the same mutation. HIV is a valid example is in this context as it is multi-strain due to the high mutation rate (Cuevas et al., Extremely High Mutation Rate of HIV-1 In Vivo. Plos Biology, 2015.). Since the proposed model is generic and not focused on COVID-19 only, it is not absolute that reinfection is even occurring. Hence, the latency periods and reinfection of the same strain are explicitly decided to not be included in the current model in order to allow it to include as many pathogens as possible since the main aim of this work is to propose a rigorous mathematical framework for multiple strains and not the best, most accurate, model for a single pathogen. That said, we would explore these suggestions in follow-up work.

Comment 2: “These issues are already considered in a recent PloS One publication of which the authors seem unaware; hence, an updated literature review is recommended.”

Answer 2: Thank you for the reference to the recent publication. We introduce new related works, including the paper (Edilson et al. 2021) proposed by the reviewer and one proposed by the second reviewer because it is very important for our research. Unfortunately, we originally submitted our manuscript before the referred works have been published and therefore were not able to address them...

Comment 3: “Observe that the model in the paper **[added by the authors: (Edilson et al., 2021)]** has a straightforward calculation of the reproduction number which essentially arrives at the same conclusion as your paper: that the reproduction number is ruled by the single strain with the highest reproduction number. Hence, that model seems richer and more useful as an off-shelf tool to predict epidemics, whilst also including optimal control.”

Answer 3: Thank you for the reference. Indeed, the works are similar in nature and it is gladdening that both Edilson et al. and us obtain a similar analytical result since this shows the robustness of the outcome in different modeling attempts. Nevertheless, the paper by Edilson et al. (2021) models dynamics in which there are n strains focused on pathogens with reinfection dynamics (please see comment #1). We would like to point out that their main difference between the two models: the proposed model takes into consideration the order of infection in the individual level allowing a complex immunity dynamic. For instance, in our model, infection in strain 1 and then strain 2 is different than strain 2 and then strain 1 in infection, recovery, and death rates. This addition generalized the SIR model in a different way than Edilson et al. and therefore richer in other cases.

Regarding the optimal control comment. Indeed, a large body of work aims to compute optimal control with a wide range of intervention protocols such as masks, lockdown, social distance and etc. While a question of interest, we believe it is out of scope for the current work since it aims to provide a realistic mathematical framework that later can be used to obtain optimal control. This claim mainly relies on the fact that the proposed model aims to be generic and an intervention policy that is appropriate to one pandemic (for instance mask-wearing for air-borne pathogens like COVID-19) will not hold for others (like HIV which requires pair-wise contact).

Comment 4: “multi-strain is an adjective, not a noun as used in the abstract.”

Answer 4: Thank you, we rephrased the abstract accordingly.

Comment 5: “there are many other papers regarding multi-strain models and optimal control which were not explored in the literature review”

Answer 5: Following this comment and other comments, we updated the introduction section, introducing four new works related to multi-strain pandemic models.

Comment 6: “experimentation with COVID data should be addressed carefully, as the data is non-standard and depends upon testing strategies and government reports, which may vary and often are way off the mark. Hence, you may be adjusting your model to a reality that does not exist”

Answer 6: Thank you for pointing it out and we completely agree with this comment. Indeed, a large number of works have been published on the subject providing a range of statistical and numerical methods to overcome this error. However, neither these works nor we are able to provide a widely-agreed baseline for the COVID-19 numbers. Moreover, a critique of the WHO historical data has been heard multiple times in the context of several governments providing inaccurate values and problematic sampling methods of the populations. Nonetheless, the WHO data is still considered a consensus on the COVID-19 and is widely used by researchers and decision-makers. It will be hard to find someone that will not agree that the COVID-19 pandemic is the most documented, measured, and open access in recorded history. Hence, it is only entreated to use this data despite the problems associated with it. To summarize, we agree with the comment, and following this comment, we better express the challenges with using COVID-19 data in Section 3.2.2.

Reviewer #2

Comment 1: “Fudolig & Howard (PLoS One, 2020) also studied a multi-strain SIR model with selective immunity through vaccination. ”

Answer 1: We thank the reviewer for bringing the newly published work to our attention. The work of Fudolig & Howard (2020) impressively explores **two-strains dynamic** and it enriched the review of works on multi-strain’s research in Section 1 (Introduction).

Comment 2: “The authors did not provide enough justification on why their work is needed”

Answer 2: Thank you for this comment. We evaluate the contribution of the proposed model in the Introduction section, second paragraph from the end.

Comment 3: “Why do we want to generalize it to M strains when it is expected that only one or two strains will be dominant in the population anyway?”

Answer 3: Following this comment, we better convey this manner in the Discussion section. We would like to shortly address this comment here as well: the motivation is two-folded. First, this is the first step in a way for a generalized SIR model with multiple strains that interchange with each other. The following step, which we are currently exploring, is replacing the strains with mutations - making it even more realistic as one can observe from the one currently taking place situation worldwide with the 4th or even 5th mutations of COVID-19. This dynamic is of course, does not unique just to COIVD-19 as other pathogens standing in the heart of other pandemics have a mutation process. For instance, HIV (Cuevas et al., Extremely High Mutation Rate of HIV-1 In Vivo. Plos Biology, 2015) or Influenza (Minayev, P., Ferguson, N., Improving the realism of deterministic multi-strain models: implications for modeling influenza A. Journal of the Royal Society Interface, 2008). Second, while pandemics are associated with pathogens, the SIR model is commonly used in rumor spread dynamics (https://www.aimsciences.org/article/doi/10.3934/dcdsb.2020124) and other network-based dynamics. For example, multiple rumors occurring at the same time and other similar scenarios can make use of such a mathematical framework.

Comment 4: “How does your model detect sharp changes in dynamics due to pandemic modifications (line 65)? Please emphasize it more in the revised manuscript.”

Answer 4: Thank you for pointing it out. After a careful review, we removed this statement from the manuscript as indeed we do not provide numerical or analytical results supporting this claim.

Comment 5: “Can you cite the source for the mean basic reproduction number, mortality rate, and a maximum number of infectious? Were these properties that you defined on your own? ”

Answer 5: Thank you for the suggestion, we cite relevant manuscripts in Section 2.1, including (Breda et al. 2021; Chatterjee et al. 2020; Baud et al. 2020; Lazebnik et al. 2021).

Comment 6: “How does the mean basic reproduction number agree with the first generation matrix approach by van den Driesche and Watmough?”

Answer 6: The proposed definition for the basic reproduction number is a forward-Euler numerical approximation to the basic reproduction number one obtains from the next generation matrix approach by van den Driesche and Watmough. The reason we use this formalization and not the one obtains from the next matrix approach is that it requires a simple computation for a specific simulation while the next-generation matrix provides a more analytical result for the dynamics and therefore is very sensitive to the model’s parameters over long periods of time such in the proposed simulations according to (Breda, 2021).

Comment 7: “Most aggressive strain: Is this an L^3 norm? ”

Answer 7: Yes, indeed. We stated it in the paper, in section 2.1.

Comment 8: “Why did you use this metric? Please elaborate.”

Answer 8: We apologize that the definition was omitted in the former version. Following this comment, we introduce in Section 2.2 a more detailed explanation for this definition. The motivation of this metric is that a higher infection rate, longer recovery rate, and higher death rate are associated with a more aggressive strain. However, due to the complexity of the pandemic spread dynamics, it is not straightforward which one of these properties is more important if any, and therefore the comparison between two strains is performed on the three properties simultaneously.

Comment 9: “Line 205 says that you assumed that no individuals recovered or died as a result of the pandemic. Then why do you have a non-zero mean mortality rate? Shouldn't this mean that your D compartment should be zero at all times?”

Answer 9: You are right - It seems to be bad wording. We mean that for the initials condition, we assume that there are no recovered or dead individuals from the pandemic as the initial condition should take place in its beginning. This statement is not referring to the pandemic dynamics. We rephrased this statement to better convey this point and now it is written like this: “ In

addition, it is assumed that no individuals have recovered or died **yet due to the pandemic at the beginning of the pandemic**”.

Comment 10: “Fudolig &Howard determined that the reproduction number of a two-strain SIR epidemic is the reproduction number of the most aggressive strain (max R_0), so it is expected for the two curves to be close. The slightly lower baseline value is only because of the averaging.”

Answer 10: Thank you for pointing it out. We introduce this explanation and extend it in the Discussion section.

Comment 11: “What is the point of modeling the curves using a logarithmic function? What does that information tell you? ”

Answer 11: The idea of fitting the dynamics is to extract a functional rule for the dynamics between the number of strains and each one of the epidemiological properties. The logarithmic function is a simple function that obtains good fitting results (R^2 = 0.79 and 0.89 for the mean basic reproduction number and the mortality rate). The logarithmic function indicates that the introduction of new strains has a reducing effect on these epidemiological properties which can be of interest to decision-makers. The fitting to the logarithmic function by itself (using the least mean square method) is not interesting but the outcome that the introduction of new mutation has a lower contribution to these epidemiological properties is of interest. Following this comment, we better convey this idea in the Discussion section and we thank the reviewer for it.

Comment 12: “In Figure 3, how do you explain the sudden rise for M=5,6?”

Answer 12: The sudden rise for M=5,6 may seem to be a bit off. Figure 3 obtained numerically and since the system is very noisy due to the large number of stochastic processes it simulates, we think the sudden rise for M=5,6 is associated with the statistical error, as one can notice by the large STD of the values. Another indication of the numerical error is the relative fitting of the logarithmic function with a coefficient of determination of 0.79. Following this comment, we aimed to better convey this point in the Discussion section.

Comment 13: "Fig. 4: Mean mortality rate graph when the authors assumed that there are no individuals who died as a result of the pandemic. If it was just a typo, how would you explain this rising mortality rate? Is it dominated by the deadliest strain (highest death rate on its own?)"

Answer 13: Thank you for drawing our attention to this abnormality. Following comment #9, it was bad wording as of course the model assumes mortality (shown both in Eqs. (1-3), Figure 1, and the numerical analysis). Therefore, a mean mortality rate graph is an entreated analysis of the proposed model.

The rising in mortality rate can be associated with the larger number of strains and therefore the larger times one can be infected and die. Moreover, as shown by Figure 3 there are just more infected individuals so there are more cases in which individuals can die. Nonetheless, these phenomena are limited, and the commelative effected reducing in the introduction of new strains - resulting in the logarithmic increasement Following this comment, we introduce these explanations to the Discussion section.

Comment 14: "Lines 265-268: The unstable equilibria provide meaning especially in multi-strain epidemics when there could be a change in dominant strain in the population. In light of recent events, it can explain the shift between an equilibrium dominated by the COVID-19 Delta variant and the COVID-19 Omicron variant. It is important to look at the stability conditions where only one strain survives."

Answer 14: Thank you for this comment. We also found this idea interesting and tried to tackle it. However, at the moment, we think the proposed model is not taking into consideration several properties of the COVID-19 pandemic like age-groups (children, adults, elderly), exposed phase, asymptomatic and symptomatic infected individuals, and much more to properly provide an explanation to the proposed query. Nonetheless, this idea is of high interest and we plan to try and tackle it in the near future with the follow-up models based on this one.

Comment 15: "Section 3.2: Can you still find the eigenvalues of the NGM for M>2 numerically? If not, this might be a good explanation to why you used the equation for the basic reproduction number in Section 2.1"

Answer 15: You are right. Below Eq. (17) we state that one cannot find using radicals (namely, via a close formula) the eigenvalues of the NGM for $|M|>2$.

One can find the eigenvalues using either numerical methods for polynomial's roots finding or eigenvalue computation such as (Lanczos, 1950) and (Trefethen and Bau, 1997). However, these methods are unstable for large polynomials and matrices which may result in large computation errors. Moreover, these methods can be highly time- and resource- consuming for a large number of strains.

Comment 16: "Figure 7: The model prediction overestimates the reproduction number after a certain time. Given how your model overestimates the WHO historical data, how useful is your model now in predicting multiple strain SIR models?"

Answer 16: Since the proposed model does not take into consideration pandemic intervention policies (i.e., lockdowns, vaccinations, etc.) we were not able to find COVID-19 related data that contains multiple strains on the one hand but does not contain pandemic intervention policies on the other hand. As such, we argue an experiment with the proposed model will not be fair. In order to tackle this challenge, in the follow-up work, we introduce a historical fitting that takes into consideration several pandemic intervention policies, which will allow us to conduct this experiment and declare the results

Comment 17: "Line 374: Where is this t-test? Which processes were compared?"

Answer 17: Thank you for pointing our attention to this - we rephrased this statement, stating the missing information and now it is written like this: "Formally, we perform a paired two-tail T-test **between the baseline and the most aggressive strain dynamics** in order to evaluate if the processes differ in a statistically significant way with \(\alpha = 0.05\) and obtain that 0 is not in the confidence interval of the statistical test".

Comment 18: "-If you used i=1 for COVID, how about i=2? Isn't the crux of the paper to be able to account for the other variants? How is this different compared to other single-strain SIR models that are more accurate in calculating the reproduction number."

Answer 18: Similar to Edilson et al. (2021), we treat the two mutations of the COVID-19 as two different strains. As such, the experiment de-facto includes two strains.

Comment 19: “I do not think that the authors worked with "the Loren Ipsum Consortium". Please revise the template file.”

Answer 19: Thank you for this comment, this is indeed a technical mistake. We removed the “Loren Ipsum Consortium” text from the paper.

Comment 20: “page 16: footnote "add here later" for the open-source code should be revised.”

Answer 20: Fixed. We introduce the code with the other supporting information according to the journal’s requirements.
