# Supplementary material for: Generic approach for mathematical model of multi-strain pandemics
Source: PLoS One. 2022 Apr 28;17(4):e0260683. doi: 10.1371/journal.pone.0260683 (PMC9049317; doi:10.1371/journal.pone.0260683)
Supplement: Attachment — Submitted filename: rebuttal_plos_one_2.pdf [file pone.0260683.s003.pdf]

First of all, we would like to thank the reviews once again for the careful review and for making sure we produce the best academic manuscript we can. It is honestly very appreciated to get such a detailed review.

Reviewer #1

Comment 1: “The comments introduced regarding the added literature are often riddled with grammar errors and seem to be rushed. Specifically, lines 97-106 are simply and candidly so badly written that the level of English is unacceptable for an academic paper; it seems the authors rushed and did not properly consider and analyze the literature. Furthermore, there are still grammar mistakes here and there, but there are many grammar mistakes introduced in the newly introduced text - a professional review is advised.”

Answer 1: Thank you for pointing it out. We took this comment very seriously and used professional language editing services to proofread our manuscript. Thus, we hope that this version of the manuscript is error-free.

Comment 2: “In Answer 1 you defend your paper by stating that it is not absolute that reinfection is even occurring, and that the objective of the model is to provide a framework for multiple strains - and not one for a single pathogen. That argument is good but is not supported by a model that is as specific as yours (single reinfection, no exposition, etc). To argue generality, your model should be the most general. For example, an SEIR model can be reduced to a SIR model and multiple reinfections from a strain can be reduced to no reinfection (just by adjusting the reinfection period to infinity). I am not suggesting that you re-do your model, but that you provide an honest discussion of the limitations when comparing to other literature.”

Answer 2: You are right - we appreciate the clarification of your previous comment. Indeed, our model can be further extended by introducing expose phase (that one can reduce for specific cases by assuming E->I rate of 1). Similarly to the SIS to SIR comparison. As such, we introduce a more complete discussion on this subject in the Introduction Section. In particular, we highlight the differences between the proposed model and other multi-strain models including the strength and limitations of the proposed model.

Comment 3: “Please include the discussion comparing your model to Arruda et al (2021) in the text. The answer regarding optimal control is not convincing

- observe that the control in Arruda et al and in other references for that matter, is not specific for COVID and does not include specific mitigation efforts. Rather, it is general and simply assesses the required level of infection prevention - that seems rather abstract and general to this reviewer. Are you suggesting that their model - which is arguably more general than yours in many aspects - though they do not consider the order of infection - is less realistic than yours? Again, please include a thorough and honest comparison in your paper."

Answer 3: The comparison between Arruda et al. (2021) and the proposed model is now provided in the Introduction Section with more details. Moreover, we extended the discussion of the usage of infection prevention in multi-strain models and clearly state that the proposed work is not handling this complexity as it would be addressed in future work based on ideas from other models such as (Reluga, 2008; Arruda et al., 2020; Edilson et al. 2021) but not limited to.

Reviewer #2

Comment 1: "Line 406: A two-tailed t-test and a confidence interval are two different things: one deals with inference and the other deals with estimation. If you want to use the result of the two-tailed t-test, then report a p-value. If you want to use the confidence interval approach, indicate that you solved for the two-sided confidence interval of the difference between the baseline and the most aggressive strain and found that zero is not included in the confidence interval, hence you can conclude that the two are sets of data are different. Bear in mind that a two-tailed t-test/two-sided confidence interval has an inequality for its alternative hypothesis. If your goal is to prove that it could be an upper limit, then maybe a one-sided t-test with a "greater than" alternative hypothesis would be more appropriate OR a one-sided confidence interval."

Answer 1: You are absolutely right - thank you very much for shedding some light on this issue. Following this comment, we recompute a one-sided confidence interval on the data to make sure the dynamics are indeed stat' different and that the most aggressive strain is an upper boundary of the baseline dynamics for our data. Following that, we alter the relevant text stating it as the reviewer suggested to make sure it is an accurate description and to allow others to easily reproduce and use our results.
